# Recent advances in pericentriolar material organization: ordered layers and scaffolding gels

**DOI:** 10.12688/f1000research.11652.1

**Published:** 2017-08-31

**Authors:** Andrew M. Fry, Josephina Sampson, Caroline Shak, Sue Shackleton

**Affiliations:** 1 Department of Molecular and Cell Biology, University of Leicester, Leicester, UK

**Keywords:** centrosomes, pericentriolar material (PCM), mitosis

## Abstract

The centrosome is an unusual organelle that lacks a surrounding membrane, raising the question of what limits its size and shape. Moreover, while electron microscopy (EM) has provided a detailed view of centriole architecture, there has been limited understanding of how the second major component of centrosomes, the pericentriolar material (PCM), is organized. Here, we summarize exciting recent findings from super-resolution fluorescence imaging, structural biology, and biochemical reconstitution that together reveal the presence of ordered layers and complex gel-like scaffolds in the PCM. Moreover, we discuss how this is leading to a better understanding of the process of microtubule nucleation, how alterations in PCM size are regulated in cycling and differentiated cells, and why mutations in PCM components lead to specific human pathologies.

## Introduction

The centrosome is a single copy organelle present in the majority of animal cells ^1^. Through concentrating proteins required for microtubule nucleation, most notably γ-tubulin and its γ-tubulin ring complex (γ-TuRC) partners, it serves as the primary microtubule organizing center (MTOC) of the cell ^2^. Centrosomes have two major structural elements: a centriole pair, consisting of two approximately 200 by 400 nm barrels that are each composed of nine highly stable microtubule triplets, and a surrounding protein-rich matrix, the PCM, that is attached to the centrioles but extends outwards to a diameter of about 1 micron. Unlike most organelles, the centrosome lacks an encompassing membrane, and although EM has shown the elegant ultrastructure of the centrioles, it has told us very little about how the PCM is organized or what defines its size and shape. Indeed, with the large coiled-coil and electron-dense nature of most PCM components, our view of the PCM has remained frustratingly opaque. Yet we have long known that the PCM is the site from which microtubules are nucleated and that microtubule nucleation capacity can be precisely modulated according to specific cues ^3^. Hence, recent technological breakthroughs in subdiffraction super-resolution imaging, together with structural biology and biochemical reconstitution approaches, now provide us with a much more detailed view of PCM architecture that is both exciting and transformative to our understanding ^4, 5^.

## The PCM proximal layer is highly organized in the interphase centrosome

Prior to super-resolution imaging, the most accurate and useful conception of the PCM was as a salt-resistant “centro-matrix” of 12–15 nm wide filaments in which circular γ-TuRCs of 25–30 nm diameter were embedded ^6– 9^. However, the development of different modalities of “optical nanoscopy” that allowed imaging below the standard diffraction limit of fluorescent light ^10^, including structured illumination microscopy (SIM), stimulated emission depletion (STED), stochastic optical-reconstruction microscopy (STORM), and photoactivated localization microscopy (PALM), led to a completely new understanding of how the PCM is organized ^4, 5^. Indeed, these super-resolution immunofluorescence microscopy approaches, mainly undertaken in *Drosophila* (fly) and human cells, have dramatically changed our perception of the PCM in interphase cells from being an amorphous mass lacking definition to a remarkably ordered assembly ^11– 14^.

**Figure 1.**
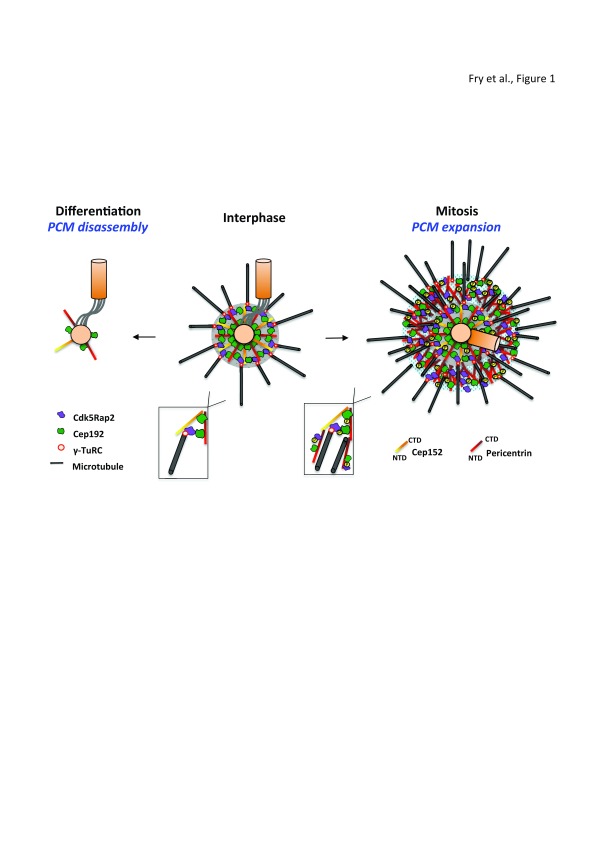
Expansion and disassembly of the PCM upon mitotic progression and differentiation. This schematic figure provides a simplified overview of PCM organization. During interphase (center panel), the two centrioles (orange cylinders) are connected by an inter-centriolar linker with the bulk of the PCM associated with the older (mother) centriole. The size and shape of the PCM is defined by the two filament proteins, pericentrin and Cep152, that extend radially from the centriole surface to generate a proximal layer (gray circle). This contains other proteins, such as Cdk5Rap2 and Cep192, which together create a branched matrix that provides binding sites for γ-TuRCs and their adaptor proteins. A second population of Cep192 is closely associated with proteins involved in centriole duplication at the centriole surface. In mitosis (right panel), PCM expansion results from the phosphorylation of multiple proteins, including pericentrin, Cdk5Rap2, and Cep192, by Plk1. This creates an outer expansive layer with gel-like properties (blue hatched circle) that is less well ordered but contains scaffolds that increase the microtubule nucleation capacity necessary for spindle assembly. Indeed, increased levels of these PCM proteins, together with additional centrosomally localized tubulin-binding proteins, such as chTOG and TPX2, may well allow microtubule nucleation to occur independently of γ-TuRCs (see inset). In contrast, in certain differentiated cells (left panel), disassembly of the proximal layer occurs with PCM proteins recruited to other non-centrosomal MTOCs. Note that the interphase centrosome is shown with two unduplicated centrioles typical of a G1 cell, whereas the mitotic centrosome contains a duplicated centriole pair that has also lost the inter-centriolar linker (mitotic cells have four centrioles, two in each spindle pole). CTD, C-terminal domain; NTD, N-terminal domain.

The first discovery using these approaches was that major components of the PCM in an interphase centrosome, including pericentrin (pericentrin-like protein [PLP] in flies), Cep152 (Asterless in flies), Cep192 (SPD-2 in flies and worms), and Cdk5Rap2 (centrosomin in flies), form annular concentric rings around the centrioles. These rings differ in diameter, ranging in cross-section from approximately 200 nm, the diameter of the centriole itself, to about 500 nm. In other words, some are located close to the centriole surface, whereas others are positioned further out. Together, these proteins form a well-organized PCM proximal layer that, from the centriole surface, extends approximately 150–200 nm in width. Importantly, using domain-specific antibodies, it was shown that for two of these proximal layer proteins, namely pericentrin and Cep152, their C-termini are closely associated with the centrioles, whereas their N-termini are further away ^11, 12, 14^. Indeed, the physical distances measured between the N- and C-termini match the predicted lengths of these elongated proteins (about 150 nm), suggesting that they form rod-like filaments with one end (C-terminus) anchored to the centriole and the other end (N-terminus) extending out towards the cytoplasm. This model provides a rationale for how these proteins can define the diameter of the PCM through acting as molecular rulers that also limit the width of the PCM proximal layer in the interphase centrosome ( Figure 1).

While this explains earlier structure–function studies on pericentrin showing that its C-terminal pericentrin-AKAP450 centrosome targeting (PACT) domain is required for centrosome binding, it remains to be determined at the molecular level how this, or the C-terminus of Cep152, interacts with centrioles ^15^. Current evidence for how the proximal layer attaches to centrioles suggests that it is likely to involve proteins that also play key roles in centriole duplication, such as CPAP (SAS-4 in flies and worms) and Plk4 ^16^. Two populations of Cep192 have also been described: one closely attached to centriole walls that contributes to both centriole duplication and attachment of the PCM proximal layer, and a second further out in the PCM proximal layer that contributes to proximal layer organization ^13, 17^. In vertebrate cells, PCM integrity is important for centriole duplication ^18^; however, this dependency may not be universal, as many species duplicate centrioles in interphase with very little PCM. Moreover, our current knowledge of how PCM proximal layer proteins functionally interact with core centriole duplication factors is sketchy at best ^1, 19, 20^. That said, genetic studies in worms recently identified a new factor, SAS-7, that potentially bridges between centrioles and SPD-2 acting upstream of both centriole duplication and PCM assembly ^21^.

The second discovery from the super-resolution microscopy studies was that, while some PCM proximal layer proteins form elongated filaments that extend from the surface of the centrioles, others are distributed amongst these filaments as, what have been termed, “branched matrix” components ^11^. These include Cdk5Rap2 and the second population of Cep192, and they depend on the filament proteins pericentrin and Cep152 for assembly into the PCM. However, the fact that pericentrin and Cep152 bind centrosomes independently suggests that there are at least two pathways for building the PCM; there is also evidence that branched matrix proteins show selectivity for particular filament proteins ^14^. Importantly, the branched matrix proteins directly anchor the γ-TuRCs, which are themselves highly organized macromolecular machines optimized for overcoming the nucleation barrier for tubulin polymerization ^2^. Furthermore, adaptor proteins are recruited, including NEDD1 and MZT1, that enable attachment of the γ-TuRC to the N-terminal centrosomin motif 1 (CM1) domain of Cdk5Rap2 while also stimulating microtubule-nucleating activity of the γ-TuRC ^22, 23^.

Through structural biology-led studies on the fly homologue centrosomin, data from the Raff lab have identified how Cdk5Rap2 can self-assemble into a branched matrix. Tetramers of centrosomin are formed via interaction of two separate dimerization motifs, a leucine zipper (LZ) in the phospho-regulated multimerization (PReM) domain at the center of the protein and the centrosomin motif 2 (CM2) domain at the C-terminus ^24, 25^. However, tetramer formation requires phosphorylation within the PReM domain by the mitotic kinase Plk1 (or Polo in flies). Without this phosphorylation, centrosomin most likely exists as a closed intramolecular homodimer, and this may explain why very little PCM is assembled around interphase centrioles in flies.

## Expansion of the mitotic PCM results from the assembly of disordered gel-like scaffolds

Super-resolution imaging suggests that, in contrast to interphase PCM, mitosis PCM is much less ordered. What has been clear for considerable time is that PCM expansion occurs through a process known as centrosome maturation that is absolutely dependent on Plk1 kinase activity, with Plk1 phosphorylating multiple PCM components, including pericentrin, Cdk5Rap2, Cep192, and, in worms, SPD-5 ^26– 30^. Worms lack pericentrin, Cep152, and Cdk5Rap2 but use the SPD-5 protein to perform analogous functions to Cdk5Rap2 and centrosomin in assembling the expansive PCM in mitosis ^28^. Plk1 phosphorylation promotes the recruitment of significantly increased amounts of these proteins, thereby creating a new expansive outer layer to the PCM that substantially increases the overall centrosome diameter.

What had been less clear until recently is the molecular basis for how phosphorylation leads to PCM expansion. However, as indicated above, structural biology studies have now revealed how the phosphorylation of centrosomin promotes the formation of inter-molecular tetramers ^25^. Plk1 phosphorylation triggers the assembly of the 2:2 complex between the CM2 and LZ domains and leads, at least *in vitro*, to the generation of micron-scale assemblies reminiscent of centrosomes. Meanwhile, biochemical reconstitution experiments with the *Caenorhabditis elegans* PCM protein, SPD-5, have led to the elegant theory that the PCM can exist without a membrane by forming a phase-separated condensate in the cytoplasm ^31^. Macromolecular crowding with agents such as polyethylene glycol drove purified SPD-5 to assemble into micron-scale assemblies that again were similar in size and shape to the PCM *in vivo*. Strikingly, these could form in suboptimal macromolecular crowding conditions if Plk1 was added, while SPD-2 also facilitated their assembly. Taken together, the current model is that Plk1 phosphorylation promotes the formation of gel-like, phase-separated condensates that, although lacking in uniform organization, most likely contain oligomeric scaffolds that enhance the capacity for microtubule nucleation.

What limits the steady-state size of this expanded mitotic PCM is likely to be a combination of a limiting cytoplasmic concentration of PCM components, together with the balance of localized Plk1 kinase activity and competing phosphatase activity. Aurora-A is another kinase that works in concert with Plk1 to promote centrosome maturation ^32^. In this case, different PCM proteins have been identified as Aurora-A substrates that directly facilitate γ-TuRC recruitment or, in the case of TACC and chTOG, stabilize microtubules. Plk1 can also phosphorylate NEDD1 either directly or indirectly via the activation of another mitotic kinase, NEK9, which increases the rate of γ-TuRC recruitment to the PCM ^30, 33, 34^. Importantly, many PCM components, including Cdk5Rap2, Cep152, and pericentrin, as well as additional effectors such as chTOG and TPX2, can directly bind α/β-tubulin heterodimers and promote microtubule nucleation independently of γ-tubulin by raising the local concentration required for spontaneous nucleation ^31, 35, 36^. Indeed, chTOG and TPX2 partition into the SPD-5 condensates generated *in vitro* using purified proteins, where they concentrate tubulin and promote microtubule nucleation in the absence of γ-tubulin ^31^.

Interestingly, the additional proteins present in the mitotic PCM do not simply stick to the outside of the PCM. In flies, centrosomin is initially recruited to the inner regions of the PCM before moving outwards in a microtubule-dependent flaring mechanism that involves flux from the inner to the outer regions of the centrosome ^37^. This recruitment to the centriole surface is dependent on the Cep192 homologue, SPD-2, while centrosomin is in turn required to maintain SPD-2 at the centrosome ^38^. This could very well be crucial to limiting the centrosome size as Plk1 is tightly localized to the centriole surface ^13^. So, as PCM proteins are recruited in mitosis, they will initially be phosphorylated by Plk1 when close to the centriole; however, this phosphorylation is gradually lost as these proteins flux outwards. Hence, assuming the competing phosphatase is evenly distributed, there will be a diminishing gradient of Plk1-mediated substrate phosphorylation as one moves out through the PCM until eventually the threshold is passed for maintaining a phase-separated condensate ^25^.

As well as undergoing maturation at mitotic onset, centrosomes execute a process known as disjunction when the duplicated pairs of centrioles lose a proteinaceous tether, or linker, that holds the two centrosomes together during interphase ^39^. This raises the question of whether this centrosome linker is part of the PCM or rather an extension of the centrioles. To answer this question, we need to understand how the molecular elements of the linker, particularly the large coiled-coil proteins C-Nap1, rootletin, and LRRC45, physically interact with well-characterized PCM and centriole proteins ^40– 42^. The linker extends between the proximal ends of the parental centrioles, and C-Nap1 can associate with the proximal-end centriole cartwheel component, Cep135. However, whether this interaction is direct or dependent on other PCM or centriole proteins is unknown. Meanwhile, rootletin has been described by immuno-EM to form an oligomeric filament that forms the major structural element of the linker, connecting the centriole proximal ends via C-Nap1 ^43, 44^. Other, smaller proteins have been identified as components of the linker, including centlein, Cep68, and β-catenin, and it will be important to explore how the linker is organized using super-resolution microscopy ^45– 47^.

Unlike the outer expansive PCM layer that is assembled in mitosis, the linker structure is disassembled upon mitotic onset as a result of phosphorylation by the NEK2, and potentially NEK5, kinase ^48– 50^. The coating of linker proteins with negative charge as a result of multi-site phosphorylation is the current favored model for linker disassembly ^39^. However, there are several lines of evidence that suggest that centrosome linker disassembly does not occur in isolation but is associated with reorganization of the PCM. Cdk5Rap2 and γ-tubulin levels are disturbed at the interphase centrosome by altered activity of NEK2 or NEK5; meanwhile, the linker protein Cep68 can interact with pericentrin and Cdk5Rap2, and loss of Cdk5Rap2 promotes premature centrosome disjunction ^47, 48, 51, 52^.

## PCM disassembly in differentiated cells

At the end of mitosis, the inactivation of Plk1 and subsequent dephosphorylation of PCM components, together with the loss of PCM fragments through flaring, lead to a return to the smaller size of PCM as defined by the proximal layer. The mechanism through which this expanded PCM is disassembled remains very poorly understood besides the requirement for the inactivation of Plk1, the dephosphorylation of Plk1 substrates, and the consequent reversal of scaffold assembly processes. Indeed, artificial tethering of Plk1 to centrioles through a PACT domain fusion prevents PCM disassembly and centrosome elimination in fly oocytes ^53^. However, there are also times in metazoan development when the proximal layer itself is disassembled under specific differentiation states and alternative MTOCs are formed in different cellular locations. A good example of this occurs during myogenesis. As myoblasts commit to differentiation, centrosome function is attenuated and microtubules instead become nucleated from the nuclear envelope, co-incident with the recruitment of PCM components to this membrane ^54– 57^. Importantly, though, the mechanisms of PCM disassembly in these specialized circumstances are currently unknown.

Considerable attention is now being turned to the questions of, first, how the PCM proximal layer is disassembled and, second, which specific PCM proteins are recruited to the non-centrosomal MTOCs to enable microtubule nucleation from these sites. Muscle cells, for example, recruit a number of PCM proteins, including pericentrin and Cdk5Rap2, to the nuclear envelope ^54, 56^. Hence, an important and intriguing question is whether a PCM-like proximal layer is assembled at non-centrosomal sites. Alternatively, these sites may assemble an oligomeric meshwork more reminiscent of the outer expansive PCM present in mitotic centrosomes. Like centrosome maturation and disjunction, the disassembly of the PCM proximal layer and then re-assembly of non-centrosomal MTOCs almost certainly depend upon post-translational modification of proximal layer proteins, although it could also involve changes in gene expression and protein degradation. In support of this, cyclin-dependent kinases regulate the formation of an MTOC at the apical membrane in *C. elegans* intestinal cells, which in turn requires SPD-2 recruitment to the membrane ^58^.

The formation of a non-centrosomal MTOC also requires a site-specific receptor for anchoring microtubule nucleation complexes in an alternative location. Recent studies have identified a muscle-specific isoform of the mammalian nuclear envelope nesprin protein family, nesprin-1α, that is required for the recruitment of pericentrin and Cdk5Rap2 as well as microtubule motor proteins to the nuclear envelope in muscle ^59, 60^. Similarly, microtubule nucleation from the Golgi apparatus occurs in various differentiated cell types ^61^. In this instance, AKAP450, which shares homology with pericentrin through the conserved C-terminal PACT domain, is necessary for microtubule nucleation at the Golgi and its recruitment is dependent upon the Golgi-specific protein GM130 ^62^. It remains to be determined, though, how these membrane proteins provide a platform for the assembly of these non-centrosomal MTOCs.

When considering the organization of non-centrosomal MTOCs, it is worth noting that land plants lack centrioles yet organize functional microtubule arrays ^63– 65^. In interphase, plants possess cortical microtubule arrays that are distributed between the plasma membrane and large internal vacuole but without a focal point of organization. These microtubules appear to be primarily nucleated from γ-tubulin-associated complexes present at the plasma membrane or on other microtubules but then self-assemble into functional arrays through conserved microtubule-associated proteins, including chTOG. However, in early mitosis, a more organized and concentrated arrangement of microtubules is somehow formed, the preprophase band; this cortical ring of microtubules assembles at the cell equator at the site of future cell division, although the underlying assembly processes are not understood. As in animal cells, nuclear envelope breakdown allows these microtubules to interact with chromatin-nucleated microtubules to form the spindle. Interestingly, as in muscle cells, microtubule nucleation occurs from the nuclear, as well as plasma, membrane in interphase ^66^. However, while γ-tubulin complexes are again implicated, current evidence suggests that these are bound to nesprin family proteins rather than orthologues of classical PCM proteins. So, in this sense, some mechanisms of non-centrosomal MTOC organization may well be conserved; however, plant cells lack homologues of pericentrin and Cdk5Rap2, suggesting that other processes are not conserved.

## Pathological consequences of PCM disruption

Increased centrosome numbers are a typical hallmark of cancer cells and can promote genomic instability through cell division errors and metastatic events through disturbance of cell polarity and migration. Meanwhile, inherited mutations in genes that encode core centriolar components can interfere with primary cilia function, causing multi-organ syndromes known as ciliopathies. In both cases, there are reasonably clear explanations for why centrosome defects contribute to disease pathology ^67, 68^. Provocatively, though, some proteins encoded by ciliopathy genes also localize to the nucleus and have roles in the DNA damage response, suggesting an alternative pathological mechanism ^69^. What is less clear is why loss-of-function mutations in PCM components underlie two forms of growth defects: one that affects the whole body, primordial dwarfism, and one that is restricted to the brain, microcephaly ^70^.

Inherited mutations in either of the two proximal layer filament proteins pericentrin or Cep152, as well as the CPAP centriole duplication factor, lead to Seckel syndrome and microcephalic osteodysplastic primordial dwarfism (MOPD) type II ^71– 74^. These primordial dwarfism syndromes exhibit profound growth retardation in every organ of the body and lead to miniature individuals. As overall size in mammals is dependent on cell number, primordial dwarfism reflects a reduction in cell number that must result from either decreased proliferation or increased cell death (or both) ^75^. The simplest explanation would be that PCM defects prevent cell cycle progression by activating cell cycle checkpoints. Indeed, there is a wealth of evidence that centrosomes act as a meeting place to facilitate many different intracellular signaling events, including checkpoint activation, that have no direct role in microtubule nucleation or organization ^76^. Moreover, as for ciliopathies, some primordial dwarfism syndromes are associated with mutations in genes that regulate the DNA damage response, and the replication stress response in particular ^75^. For example, PCM proteins are necessary to recruit ATR, a key checkpoint protein that monitors replication fork integrity, to the centrosome to facilitate DNA damage signaling and loss-of-function ATR mutations also lead to Seckel syndrome ^77^. Hence, in the absence of an intact centrosome, cell cycle progression halts.

An alternative explanation is that defects prevent the PCM expansion necessary for proper spindle assembly and mitotic progression, thus leading to mitotic catastrophe and increasing cell death. However, a major conundrum in explaining primordial dwarfism is that these processes would have to affect all organs equally. It is possible that a uniform effect on stem cells during early development could cause a similar reduction in the progenitor pools that control particular tissue sizes. Close-range homeostatic mechanisms might also ensure an appropriate balance in the sizes of neighboring tissues, while more long-range effects could be exerted through endocrine organs that control tissue growth throughout the body. However, these are just ideas, and it is safe to say that we remain far from understanding the pathological basis for why PCM defects cause primordial dwarfism.

In contrast to primordial dwarfism, microcephaly is a specific reduction in brain size without affecting other organs ^78^. Intriguingly, the majority of genes implicated in autosomal recessive primary microcephaly encode centrosomal proteins, including both centriolar and PCM components ^67, 70^. Notably, a different set of mutations in Cep152 cause microcephaly to those that cause Seckel syndrome ^79^, while mutations in Cdk5Rap2 were amongst the first to be identified in microcephaly patients ^80^. The current hypothesis for why centrosomal proteins are particularly prominent in this disease focuses very much on the disturbance of cell divisions within the neural progenitor pool. These undergo not only symmetric divisions in early development to expand the pool but also asymmetric divisions to generate differentiated neurons whilst concomitantly replenishing the progenitor pool. As with primordial dwarfism, PCM defects that interfere with cell cycle progression by activating checkpoints or induce cell death as a result of mitotic defects would explain the reduced neural progenitor pool. Indeed, mutations in other centrosomal genes that cause primary microcephaly, such as *STIL*, result in centriole amplification ^67^, and the experimental induction of centriole amplification by overexpression of Plk4 leads to a microcephalic condition in mice primarily through mitotic errors and excessive apoptosis ^81^. Equally, though, errors in spindle positioning that prevent asymmetric divisions would cause failure to either generate differentiated cells or replenish the progenitor pool. The organ-specific nature of this developmental disease does strongly suggest that neural progenitors are exquisitely sensitive to perturbations in these processes, and one can speculate that this is due to the heavy reliance on asymmetric divisions to generate large brains.

## Future perspectives

The combination of super-resolution microscopy, structural biology, and biochemical reconstitution is beginning to provide the long-sought-after mechanistic details of how the PCM is organized and regulated. It has also revealed important similarities and differences between species. The PCM proximal layer present in interphase centrosomes is largely assembled around pericentrin/PLP and Cep152/Asterless in mammals and flies. However, neither of these proteins is found in worms. Mammals, flies, and worms all have a Cep192/SPD-2 protein that, at least in mammals, aids the connection between the centrioles and proximal layer. However, the amount of PCM present in interphase in flies and worms appears to be minimal, and it is not clear whether SPD-2 has a role at this time. In the expanded mitotic PCM, mammals and flies use the related Cdk5Rap2 and centrosomin proteins to form extended scaffolds, while this function is performed in worms by SPD-5 that, at least by sequence, is unrelated. Cep192/SPD-2 have a more obviously conserved role across all species in the expanded mitotic PCM. Indeed, the principle of an ordered proximal layer at the PCM when present in interphase and a more disordered gel-like scaffold in the expanded PCM in mitosis does appear to be universally shared.

These findings are stimulating specific structure–function studies into, for example, how the proximal layer regulates centrosome size, how the filament proteins are anchored to centriole walls, and how expansion and disassembly of the PCM are regulated by post-translational modifications. Relevant to this is whether the electron-dense centriolar satellites that surround centrosomes in some vertebrate cells represent supra-assemblies of PCM complexes that are being trafficked to centrosomes. Indeed, understanding the functional relationship between centriolar satellites and the PCM should explain whether there are proteins, such as PCM-1, that have their primary role in centriolar satellite integrity and why proteins implicated in ciliopathies seem to be over-represented in centriolar satellites ^82, 83^.

Unfortunately, apart from the importance of Plk1, we still know little about the molecular events that control PCM expansion, disassembly, and relocation. A biochemical understanding of the roles of individual sites phosphorylated by Plk1, and Aurora-A for that matter, will be required as well as identification of the competing phosphatases. The recent demonstration of how Plk1 phosphorylation of *Drosophila* Cdk5Rap2 may promote oligomerization through exposing a dimerization interface is an excellent exemplar ^25^. However, other types of modification will almost certainly contribute to this, including, for example, the acetylation or ubiquitylation of PCM proteins. It is also not clear how pericentrin, which binds centrioles through its PACT domain to create the proximal layer in interphase, is recruited to the outer layer during PCM expansion in mitosis ^84^. Equally, the need to understand mechanisms that drive PCM disassembly is highlighted by the finding that formation of the nuclear MTOC in myotubes is disrupted in certain forms of muscular dystrophy and may contribute to disease pathophysiology ^85^.

Perhaps unexpectedly, we have developed a reasonably coherent model for PCM assembly and recruitment of γ-TuRCs based on just a handful of proteins. Yet more than 100 proteins have been described as PCM components; so what are the rest doing? On one hand, there is good evidence, as indicated earlier, that the centrosome can act as a meeting place for signaling proteins that are not directly involved in microtubule organization. However, ruling out a role for a particular PCM protein in microtubule organization is not easy. In this regard, the *in vitro* reconstitution of PCM assembly will be a particularly valuable approach and should identify the minimal set of components required for different PCM functions, including efficient microtubule nucleation and anchoring.

Finally, we need to understand how genetic mutations that affect PCM components give rise to growth-related pathologies. Our current knowledge can broadly explain the reduction in cell number, be it from checkpoint-mediated cell cycle arrest, increased cell death resulting from mitotic errors, or spindle orientation defects that perturb stem cell pools. However, the uniformity of organs affected in primordial dwarfism and, conversely, the tissue specificity in primary microcephaly are striking and difficult to explain, particularly considering that different mutations in the same protein (Cep152) can give rise to one or other pathology. Answering these questions will come at least in part from complementing what we have learnt from super-resolution, structural biology, and biochemical reconstitution studies with gene-editing approaches not just in cells but also in whole animals.

## Abbreviations

CM1 and 2, centrosomin motif 1 and 2; EM, electron microscopy; LZ, leucine zipper; MTOC, microtubule organizing center; PACT, pericentrin-AKAP450 centrosome targeting; PCM, pericentriolar material; PLP, pericentrin-like protein; PReM, phospho-regulated multimerization domain; γ-TuRC, γ-tubulin ring complex.

## References

[1] Conduit PT Wainman A Raff JW: Centrosome function and assembly in animal cells. *Nat Rev Mol Cell Biol.* 2015;16(10):611–24. 10.1038/nrm4062 26373263

[2] Kollman JM Merdes A Mourey L: Microtubule nucleation by γ-tubulin complexes. *Nat Rev Mol Cell Biol.* 2011;12(11):709–21. 10.1038/nrm3209 21993292 PMC7183383

[3] Gould RR Borisy GG: The pericentriolar material in Chinese hamster ovary cells nucleates microtubule formation. *J Cell Biol.* 1977;73(3):601–15. 10.1083/jcb.73.3.601 559676 PMC2111414

[4] Mennella V Agard DA Huang B: Amorphous no more: subdiffraction view of the pericentriolar material architecture. *Trends Cell Biol.* 2014;24(3):188–97. 10.1016/j.tcb.2013.10.001 24268653 PMC3991556

[5] Woodruff JB Wueseke O Hyman AA: Pericentriolar material structure and dynamics. *Philos Trans R Soc Lond B Biol Sci.* 2014;369(1650): pii: 20130459. 10.1098/rstb.2013.0459 25047613 PMC4113103

[6] Moritz M Zheng Y Alberts BM: Recruitment of the gamma-tubulin ring complex to *Drosophila* salt-stripped centrosome scaffolds. *J Cell Biol.* 1998;142(3):775–86. 10.1083/jcb.142.3.775 9700165 PMC2148159

[7] Dictenberg JB Zimmerman W Sparks CA: Pericentrin and gamma-tubulin form a protein complex and are organized into a novel lattice at the centrosome. *J Cell Biol.* 1998;141(1):163–74. 10.1083/jcb.141.1.163 9531556 PMC2132723

[8] Schnackenberg BJ Hull DR Balczon RD: Reconstitution of microtubule nucleation potential in centrosomes isolated from Spisula solidissima oocytes. *J Cell Sci.* 2000;113(Pt 6):943–53. 10683143 10.1242/jcs.113.6.943

[9] Paintrand M Moudjou M Delacroix H: Centrosome organization and centriole architecture: their sensitivity to divalent cations. *J Struct Biol.* 1992;108(2):107–28. 10.1016/1047-8477(92)90011-X 1486002

[10] Huang B Babcock H Zhuang X: Breaking the diffraction barrier: super-resolution imaging of cells. *Cell.* 2010;143(7):1047–58. 10.1016/j.cell.2010.12.002 21168201 PMC3272504

[11] Mennella V Keszthelyi B McDonald KL: Subdiffraction-resolution fluorescence microscopy reveals a domain of the centrosome critical for pericentriolar material organization. *Nat Cell Biol.* 2012;14(11):1159–68. 10.1038/ncb2597 23086239 PMC3767400

[12] Sonnen KF Schermelleh L Leonhardt H: 3D-structured illumination microscopy provides novel insight into architecture of human centrosomes. *Biol Open.* 2012;1(10):965–76. 10.1242/bio.20122337 23213374 PMC3507176

[13] Fu J Glover DM: Structured illumination of the interface between centriole and peri-centriolar material. *Open Biol.* 2012;2(8):120104. 10.1098/rsob.120104 22977736 PMC3438536

[14] Lawo S Hasegan M Gupta GD: Subdiffraction imaging of centrosomes reveals higher-order organizational features of pericentriolar material. *Nat Cell Biol.* 2012;14(11):1148–58. 10.1038/ncb2591 23086237

[15] Gillingham AK Munro S: The PACT domain, a conserved centrosomal targeting motif in the coiled-coil proteins AKAP450 and pericentrin. *EMBO Rep.* 2000;1(6):524–9. 10.1093/embo-reports/kvd105 11263498 PMC1083777

[16] Cizmecioglu O Arnold M Bahtz R: Cep152 acts as a scaffold for recruitment of Plk4 and CPAP to the centrosome. *J Cell Biol.* 2010;191(4):731–9. 10.1083/jcb.201007107 21059844 PMC2983070

[17] Sonnen KF Gabryjonczyk AM Anselm E: Human Cep192 and Cep152 cooperate in Plk4 recruitment and centriole duplication. *J Cell Sci.* 2013;126(Pt 14):3223–33. 10.1242/jcs.129502 23641073

[18] Loncarek J Hergert P Magidson V: Control of daughter centriole formation by the pericentriolar material. *Nat Cell Biol.* 2008;10(3):322–8. 10.1038/ncb1694 18297061 PMC2365476

[19] Nigg EA Stearns T: The centrosome cycle: Centriole biogenesis, duplication and inherent asymmetries. *Nat Cell Biol.* 2011;13(10):1154–60. 10.1038/ncb2345 21968988 PMC3947860

[20] Gönczy P: Towards a molecular architecture of centriole assembly. *Nat Rev Mol Cell Biol.* 2012;13(7):425–35. 10.1038/nrm3373 22691849

[21] Sugioka K Hamill DR Lowry JB: Centriolar SAS-7 acts upstream of SPD-2 to regulate centriole assembly and pericentriolar material formation. *eLife.* 2017;6: pii: e20353. 10.7554/eLife.20353 28092264 PMC5342823

[22] Cota RR Teixidó-Travesa N Ezquerra A: MZT1 regulates microtubule nucleation by linking γTuRC assembly to adapter-mediated targeting and activation. *J Cell Sci.* 2017;130(2):406–19. 10.1242/jcs.195321 27852835

[23] Lin TC Neuner A Flemming D: MOZART1 and γ-tubulin complex receptors are both required to turn γ-TuSC into an active microtubule nucleation template. *J Cell Biol.* 2016;215(6):823–40. 10.1083/jcb.201606092 27920216 PMC5166503

[24] Conduit PT Feng Z Richens JH: The centrosome-specific phosphorylation of Cnn by Polo/Plk1 drives Cnn scaffold assembly and centrosome maturation. *Dev Cell.* 2014;28(6):659–69. 10.1016/j.devcel.2014.02.013 24656740 PMC3988887

[25] Feng Z Caballe A Wainman A: Structural Basis for Mitotic Centrosome Assembly in Flies. *Cell.* 2017;169(6):1078–1089.e13. 10.1016/j.cell.2017.05.030 28575671 PMC5457487

[26] Lee K Rhee K: PLK1 phosphorylation of pericentrin initiates centrosome maturation at the onset of mitosis. *J Cell Biol.* 2011;195(7):1093–101. 10.1083/jcb.201106093 22184200 PMC3246884

[27] Dobbelaere J Josué F Suijkerbuijk S: A genome-wide RNAi screen to dissect centriole duplication and centrosome maturation in *Drosophila*. *PLoS Biol.* 2008;6(9):e224. 10.1371/journal.pbio.0060224 18798690 PMC2535660

[28] Woodruff JB Wueseke O Viscardi V: Centrosomes. Regulated assembly of a supramolecular centrosome scaffold *in vitro*. *Science.* 2015;348(6236):808–12. 10.1126/science.aaa3923 25977552 PMC5039038

[29] Mahen R Jeyasekharan AD Barry NP: Continuous polo-like kinase 1 activity regulates diffusion to maintain centrosome self-organization during mitosis. *Proc Natl Acad Sci U S A.* 2011;108(22):9310–5. 10.1073/pnas.1101112108 21576470 PMC3107272

[30] Haren L Stearns T Lüders J: Plk1-dependent recruitment of gamma-tubulin complexes to mitotic centrosomes involves multiple PCM components. *PLoS One.* 2009;4(6):e5976. 10.1371/journal.pone.0005976 19543530 PMC2695007

[31] Woodruff JB Ferreira Gomes B Widlund PO: The Centrosome Is a Selective Condensate that Nucleates Microtubules by Concentrating Tubulin. *Cell.* 2017;169(6):1066–1077.e10. 10.1016/j.cell.2017.05.028 28575670

[32] Asteriti IA De Mattia F Guarguaglini G: Cross-Talk between AURKA and Plk1 in Mitotic Entry and Spindle Assembly. *Front Oncol.* 2015;5:283. 10.3389/fonc.2015.00283 26779436 PMC4688340

[33] Sdelci S Schütz M Pinyol R: Nek9 phosphorylation of NEDD1/GCP-WD contributes to Plk1 control of γ-tubulin recruitment to the mitotic centrosome. *Curr Biol.* 2012;22(16):1516–23. 10.1016/j.cub.2012.06.027 22818914

[34] Gomez-Ferreria MA Bashkurov M Helbig AO: Novel NEDD1 phosphorylation sites regulate γ-tubulin binding and mitotic spindle assembly. *J Cell Sci.* 2012;125(Pt 16):3745–51. 10.1242/jcs.105130 22595525

[35] Roostalu J Cade NI Surrey T: Complementary activities of TPX2 and chTOG constitute an efficient importin-regulated microtubule nucleation module. *Nat Cell Biol.* 2015;17(11):1422–34. 10.1038/ncb3241 26414402 PMC4826748

[36] Wieczorek M Bechstedt S Chaaban S: Microtubule-associated proteins control the kinetics of microtubule nucleation. *Nat Cell Biol.* 2015;17(7):907–16. 10.1038/ncb3188 26098575

[37] Conduit PT Brunk K Dobbelaere J: Centrioles regulate centrosome size by controlling the rate of Cnn incorporation into the PCM. *Curr Biol.* 2010;20(24):2178–86. 10.1016/j.cub.2010.11.011 21145741

[38] Conduit PT Richens JH Wainman A: A molecular mechanism of mitotic centrosome assembly in *Drosophila*. *eLife.* 2014;3:e03399. 10.7554/eLife.03399 25149451 PMC4175739

[39] Mardin BR Schiebel E: Breaking the ties that bind: new advances in centrosome biology. *J Cell Biol.* 2012;197(1):11–8. 10.1083/jcb.201108006 22472437 PMC3317805

[40] Fry AM Mayor T Meraldi P: C-Nap1, a novel centrosomal coiled-coil protein and candidate substrate of the cell cycle-regulated protein kinase Nek2. *J Cell Biol.* 1998;141(7):1563–74. 10.1083/jcb.141.7.1563 9647649 PMC2133000

[41] Yang J Adamian M Li T: Rootletin interacts with C-Nap1 and may function as a physical linker between the pair of centrioles/basal bodies in cells. *Mol Biol Cell.* 2006;17(2):1033–40. 10.1091/mbc.E05-10-0943 16339073 PMC1356609

[42] He R Huang N Bao Y: LRRC45 is a centrosome linker component required for centrosome cohesion. *Cell Rep.* 2013;4(6):1100–7. 10.1016/j.celrep.2013.08.005 24035387

[43] Hardy T Lee M Hames RS: Multisite phosphorylation of C-Nap1 releases it from Cep135 to trigger centrosome disjunction. *J Cell Sci.* 2014;127(Pt 11):2493–506. 10.1242/jcs.142331 24695856 PMC4038944

[44] Bahe S Stierhof YD Wilkinson CJ: Rootletin forms centriole-associated filaments and functions in centrosome cohesion. *J Cell Biol.* 2005;171(1):27–33. 10.1083/jcb.200504107 16203858 PMC2171225

[45] Bahmanyar S Kaplan DD Deluca JG: beta-Catenin is a Nek2 substrate involved in centrosome separation. *Genes Dev.* 2008;22(1):91–105. 10.1101/gad.1596308 18086858 PMC2151018

[46] Fang G Zhang D Yin H: Centlein mediates an interaction between C-Nap1 and Cep68 to maintain centrosome cohesion. *J Cell Sci.* 2014;127(Pt 8):1631–9. 10.1242/jcs.139451 24554434

[47] Graser S Stierhof YD Nigg EA: Cep68 and Cep215 (Cdk5rap2) are required for centrosome cohesion. *J Cell Sci.* 2007;120(Pt 24):4321–31. 10.1242/jcs.020248 18042621

[48] Prosser SL Sahota NK Pelletier L: Nek5 promotes centrosome integrity in interphase and loss of centrosome cohesion in mitosis. *J Cell Biol.* 2015;209(3):339–48. 10.1083/jcb.201412099 25963817 PMC4427792

[49] Cervenka I Valnohova J Bernatik O: Dishevelled is a NEK2 kinase substrate controlling dynamics of centrosomal linker proteins. *Proc Natl Acad Sci U S A.* 2016;113(33):9304–9. 10.1073/pnas.1608783113 27486244 PMC4995965

[50] Faragher AJ Fry AM: Nek2A kinase stimulates centrosome disjunction and is required for formation of bipolar mitotic spindles. *Mol Biol Cell.* 2003;14(7):2876–89. 10.1091/mbc.E03-02-0108 12857871 PMC165683

[51] Barrera JA Kao LR Hammer RE: CDK5RAP2 regulates centriole engagement and cohesion in mice. *Dev Cell.* 2010;18(6):913–26. 10.1016/j.devcel.2010.05.017 20627074 PMC3078807

[52] Pagan JK Marzio A Jones MJ: Degradation of Cep68 and PCNT cleavage mediate Cep215 removal from the PCM to allow centriole separation, disengagement and licensing. *Nat Cell Biol.* 2015;17(1):31–43. 10.1038/ncb3076 25503564 PMC4415623

[53] Pimenta-Marques A Bento I Lopes CA: A mechanism for the elimination of the female gamete centrosome in *Drosophila* melanogaster. *Science.* 2016;353(6294):aaf4866. 10.1126/science.aaf4866 27229142

[54] Bugnard E Zaal KJ Ralston E: Reorganization of microtubule nucleation during muscle differentiation. *Cell Motil Cytoskeleton.* 2005;60(1):1–13. 10.1002/cm.20042 15532031

[55] Connolly JA Kiosses BW Kalnins VI: Centrioles are lost as embryonic myoblasts fuse into myotubes *in vitro*. *Eur J Cell Biol.* 1986;39(2):341–5. 3514220

[56] Srsen V Fant X Heald R: Centrosome proteins form an insoluble perinuclear matrix during muscle cell differentiation. *BMC Cell Biol.* 2009;10:28. 10.1186/1471-2121-10-28 19383121 PMC2676252

[57] Tassin AM Maro B Bornens M: Fate of microtubule-organizing centers during myogenesis *in vitro*. *J Cell Biol.* 1985;100(1):35–46. 10.1083/jcb.100.1.35 3880758 PMC2113478

[58] Yang R Feldman JL: SPD-2/CEP192 and CDK Are Limiting for Microtubule-Organizing Center Function at the Centrosome. *Curr Biol.* 2015;25(14):1924–31. 10.1016/j.cub.2015.06.001 26119750

[59] Espigat-Georger A Dyachuk V Chemin C: Nuclear alignment in myotubes requires centrosome proteins recruited by nesprin-1. *J Cell Sci.* 2016;129(22):4227–37. 10.1242/jcs.191767 27802164

[60] Holt I Duong NT Zhang Q: Specific localization of nesprin-1-α2, the short isoform of nesprin-1 with a KASH domain, in developing, fetal and regenerating muscle, using a new monoclonal antibody. *BMC Cell Biol.* 2016;17(1):26. 10.1186/s12860-016-0105-9 27350129 PMC4924313

[61] Efimov A Kharitonov A Efimova N: Asymmetric CLASP-dependent nucleation of noncentrosomal microtubules at the *trans*-Golgi network. *Dev Cell.* 2007;12(6):917–30. 10.1016/j.devcel.2007.04.002 17543864 PMC2705290

[62] Rivero S Cardenas J Bornens M: Microtubule nucleation at the cis-side of the Golgi apparatus requires AKAP450 and GM130. *EMBO J.* 2009;28(8):1016–28. 10.1038/emboj.2009.47 19242490 PMC2683699

[63] Zhang H Dawe RK: Mechanisms of plant spindle formation. *Chromosome Res.* 2011;19(3):335–44. 10.1007/s10577-011-9190-y 21424324

[64] Hamada T: Microtubule organization and microtubule-associated proteins in plant cells. *Int Rev Cell Mol Biol.* 2014;312:1–52. 10.1016/B978-0-12-800178-3.00001-4 25262237

[65] Yamada M Goshima G: Mitotic Spindle Assembly in Land Plants: Molecules and Mechanisms. *Biology (Basel).* 2017;6(1): pii: E6. 10.3390/biology6010006 28125061 PMC5371999

[66] Janski N Masoud K Batzenschlager M: The GCP3-interacting proteins GIP1 and GIP2 are required for γ-tubulin complex protein localization, spindle integrity, and chromosomal stability. *Plant Cell.* 2012;24(3):1171–87. 10.1105/tpc.111.094904 22427335 PMC3336128

[67] Nigg EA Čajánek L Arquint C: The centrosome duplication cycle in health and disease. *FEBS Lett.* 2014;588(15):2366–72. 10.1016/j.febslet.2014.06.030 24951839

[68] Nigg EA Raff JW: Centrioles, centrosomes, and cilia in health and disease. *Cell.* 2009;139(4):663–78. 10.1016/j.cell.2009.10.036 19914163

[69] Johnson CA Collis SJ: Ciliogenesis and the DNA damage response: a stressful relationship. *Cilia.* 2016;5:19. 10.1186/s13630-016-0040-6 27335639 PMC4916530

[70] Chavali PL Pütz M Gergely F: Small organelle, big responsibility: the role of centrosomes in development and disease. *Philos Trans R Soc Lond B Biol Sci.* 2014;369(1650): pii: 20130468. 10.1098/rstb.2013.0468 25047622 PMC4113112

[71] Kalay E Yigit G Aslan Y: CEP152 is a genome maintenance protein disrupted in Seckel syndrome. *Nat Genet.* 2011;43(1):23–6. 10.1038/ng.725 21131973 PMC3430850

[72] Rauch A Thiel CT Schindler D: Mutations in the pericentrin ( *PCNT*) gene cause primordial dwarfism. *Science.* 2008;319(5864):816–9. 10.1126/science.1151174 18174396

[73] Griffith E Walker S Martin CA: Mutations in pericentrin cause Seckel syndrome with defective ATR-dependent DNA damage signaling. *Nat Genet.* 2008;40(2):232–6. 10.1038/ng.2007.80 18157127 PMC2397541

[74] Al-Dosari MS Shaheen R Colak D: Novel CENPJ mutation causes Seckel syndrome. *J Med Genet.* 2010;47(6):411–4. 10.1136/jmg.2009.076646 20522431

[75] Klingseisen A Jackson AP: Mechanisms and pathways of growth failure in primordial dwarfism. *Genes Dev.* 2011;25(19):2011–24. 10.1101/gad.169037 21979914 PMC3197200

[76] Arquint C Gabryjonczyk AM Nigg EA: Centrosomes as signalling centres. *Philos Trans R Soc Lond B Biol Sci.* 2014;369(1650): pii: 20130464. 10.1098/rstb.2013.0464 25047618 PMC4113108

[77] Tibelius A Marhold J Zentgraf H: Microcephalin and pericentrin regulate mitotic entry via centrosome-associated Chk1. *J Cell Biol.* 2009;185(7):1149–57. 10.1083/jcb.200810159 19546241 PMC2712957

[78] Thornton GK Woods CG: Primary microcephaly: do all roads lead to Rome? *Trends Genet.* 2009;25(11):501–10. 10.1016/j.tig.2009.09.011 19850369 PMC2816178

[79] Guernsey DL Jiang H Hussin J: Mutations in centrosomal protein CEP152 in primary microcephaly families linked to MCPH4. *Am J Hum Genet.* 2010;87(1):40–51. 10.1016/j.ajhg.2010.06.003 20598275 PMC2896783

[80] Bond J Roberts E Springell K: A centrosomal mechanism involving CDK5RAP2 and CENPJ controls brain size. *Nat Genet.* 2005;37(4):353–5. 10.1038/ng1539 15793586

[81] Marthiens V Rujano MA Pennetier C: Centrosome amplification causes microcephaly. *Nat Cell Biol.* 2013;15(7):731–40. 10.1038/ncb2746 23666084

[82] Bärenz F Mayilo D Gruss OJ: Centriolar satellites: busy orbits around the centrosome. *Eur J Cell Biol.* 2011;90(12):983–9. 10.1016/j.ejcb.2011.07.007 21945726

[83] Lopes CA Prosser SL Romio L: Centriolar satellites are assembly points for proteins implicated in human ciliopathies, including oral-facial-digital syndrome 1. *J Cell Sci.* 2011;124(Pt 4):600–12. 10.1242/jcs.077156 21266464 PMC3031371

[84] Kim S Rhee K: Importance of the CEP215-pericentrin interaction for centrosome maturation during mitosis. *PLoS One.* 2014;9(1):e87016. 10.1371/journal.pone.0087016 24466316 PMC3899370

[85] Meinke P Mattioli E Haque F: Muscular dystrophy-associated *SUN1* and *SUN2* variants disrupt nuclear-cytoskeletal connections and myonuclear organization. *PLoS Genet.* 2014;10(9):e1004605. 10.1371/journal.pgen.1004605 25210889 PMC4161305

